# Interaction between Gallotannin and a Recombinant Form of Arginine Kinase of *Trypanosoma brucei*: Thermodynamic and Spectrofluorimetric Evaluation

**DOI:** 10.1155/2014/675905

**Published:** 2014-08-26

**Authors:** O. S. Adeyemi, A. F. Sulaiman, O. M. Iniaghe

**Affiliations:** ^1^Department of Biological Sciences, Landmark University, Omu-Aran 370102, Nigeria; ^2^Department of Biochemistry, Faculty of Life Sciences, University of Ilorin, Ilorin 240001, Nigeria; ^3^Department of Biochemistry, Ambrose Alli University, Ekpoma 310001, Nigeria

## Abstract

Current chemotherapies against trypanosomiasis are beset with diverse challenges, a situation which underscores the numerous research efforts aimed at finding newer and effective treatments. Arginine kinase of trypanosome has been validated as target for drug development against trypanosomiasis. The present study investigated the interaction between a recombinant form of the arginine kinase (rTbAK) of trypanosome and gallotannin. The interaction between gallotannin and recombinant arginine kinase of* Trypanosoma brucei* caused significant decrease of enzyme activity. Kinetic analysis revealed the interaction to be of noncompetitive inhibition. Further thermodynamic analysis showed that the interaction between gallotannin and the recombinant arginine kinase was nonspontaneous and involved hydrophobic forces. The *K*_sv_ values and the FRET analysis suggest that static quenching of fluorescence intensity by gallotannin was static. Data revealed inhibitory interactions between gallotannin and rTbAK of trypanosome. Although the mechanism of inhibition is not clear yet, molecular docking studies are ongoing to clearly define the inhibitory interactions between the gallotannin and rTbAK. The knowledge of such binding properties would enrich development of selective inhibitors for the arginine kinase of* Trypanosoma brucei*.

## 1. Introduction

African trypanosomiasis is one of the neglected diseases currently ravaging several countries in the sub-Saharan Africa. The disease, which affects both humans and animals, is fatal if left untreated. Chemotherapy which forms the major means of controlling the disease scourge is currently faced with many challenges including limited efficacy, unwanted toxicity, and emergence of resistant strain of trypanosomes [1]. These and other factors underscore the research efforts aimed at finding better chemotherapy for trypanosomiasis. Arginine kinase (AK) is a phosphotransferase enzyme, which has been validated as a drug target for selective trypanocide development [2–5]. The enzyme mediates the reversible formation of a phosphagen from L-arginine and ATP [2]. Phosphoarginine plays key role in providing the needed energy for cellular activity until metabolic events such as glycolysis and oxidative phosphorylation are switched on [6–8]. In a separate study, it has been demonstrated that AK is highly critical to the survival of the trypanosomes under stressful condition, particularly for the bloodstream forms of trypanosome, which are constantly being exposed to prooxidants in the mammalian host blood environment [9]. The AK could serve as ready source, not only of ATP, but also of inorganic phosphate that is highly critical for active metabolic processes [5]. Therefore, reduction or absence of AK activity would present dire consequences to the growth and survival of the parasites. In light of this, compounds that selectively inhibit the AK enzyme are desirable as such compounds could become lead candidates for early development of trypanocides. More so, AK is absent in humans, thus making it a choice target for selective inhibition as well as for trypanocide development [10].

Gallotannin belongs to the group of polyphenolic compounds which in earlier studies have been implicated for antitrypanosomal activities attributable to their iron chelating potential [11]. Furthermore, earlier studies have attributed the trypanolytic potential of some plant extracts to the presence of polyphenolic compounds [1] and [12]. It is, therefore, plausible to evaluate compounds with trypanolytic potential for activity against identified trypanosomal enzyme targets.

In the light of this, the present study sought to investigate the interaction between gallotannin and a recombinant form of arginine kinase (rTbAK) enzyme obtained from* T. brucei*.

## 2. Results

### 2.1. Estimation of Kinetic Parameters for the Interaction between Gallotannin and the Recombinant Arginine Kinase of* Trypanosoma brucei* (rTbAK) Protein

**Figure 1:**
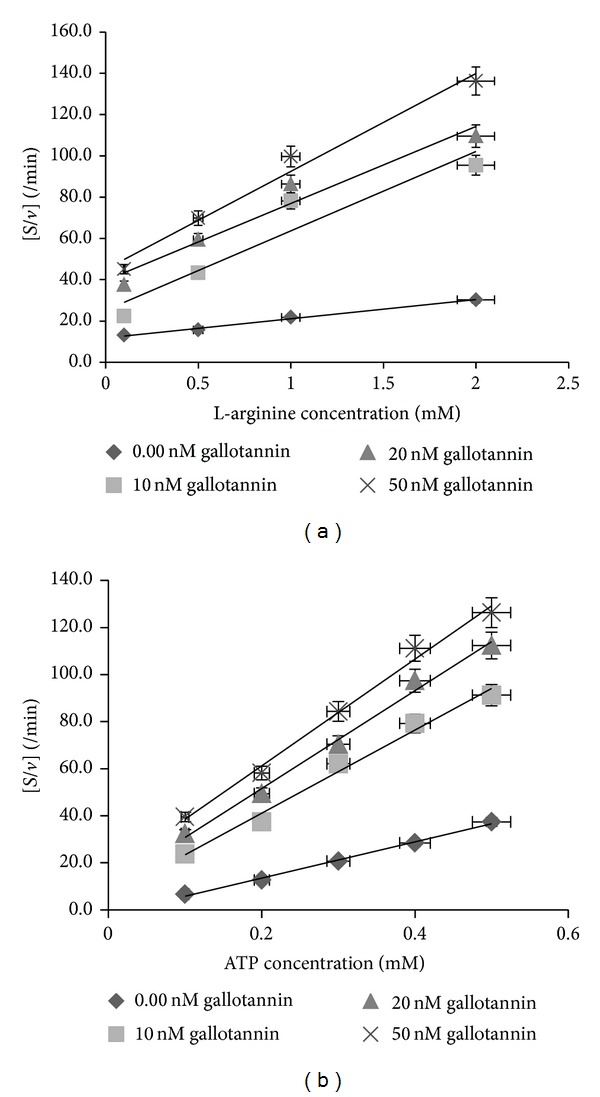
Hanes-Woolf plots for the interaction of gallotannin with a recombinant arginine kinase of* Trypanosoma brucei* in the presence of different concentrations of (a) L-arginine and (b) ATP, respectively.

**Figure 2:**
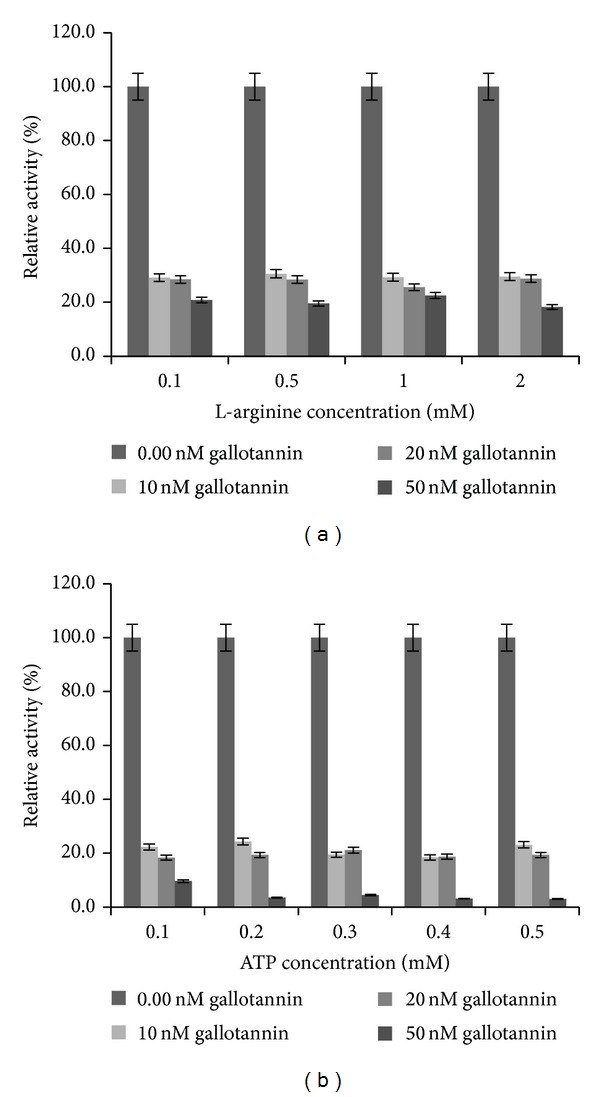
Relative activities of recombinant arginine kinase of* Trypanosoma brucei* in the presence of gallotannin at different concentrations of (a) L-arginine and (b) ATP.

The interaction of various concentrations of gallotannin resulted in significant decrease in the activity of rTbAK by an approximate >80% relative to the control assay, which lacked the presence of gallotannin (Figures 1 and 2). The kinetic parameters for the interaction of gallotannin with rTbAK were determined by including the gallotannin at a concentration of 10–50 nM with either arginine, between 0.1 and 2 mM concentration at fixed level of ATP (0.5 mM), or ATP, between 0.1 and 0.5 mM concentration at fixed level of arginine (2 mM). The results showed that, with respect to arginine as a variable substrate, the *V*_max⁡_ and *K*_*m*_ are 0.120 *μ*mol/min/mL and 1.28 nM, respectively. Results showed that the *V*_max⁡_ decreased while the *K*_*m*_ virtually remained unaffected. At the concentration 50 nM, gallotannin decreased the *V*_max⁡_ from 0.120 *μ*mol/min/mL to 0.020 *μ*mol/min/mL representing >84% decrease. At concentrations 10 and 20 nM of gallotannin, rTBAK had *V*_max⁡_^app^ values as 0.026 and 0.027 *μ*mol/min/mL, respectively.

With respect to ATP as a variable substrate, the *V*_max⁡_ and *K*_*m*_ are 0.13 *μ*mol/min/mL and 0.26 mM, respectively. At the concentration 50 nM of gallotannin, the rTBAK had *V*_max⁡_^app^ value 0.004 *μ*mol/min/mL approximately 97% decrease. At concentrations 10 and 20 nM of gallotannin, the rTBAK had >90% decrease in *V*_max⁡_.

In order to evaluate the binding affinity of rTBAK for gallotannin, the inhibitor constant (*K*_*i*_) was estimated using (1). Results showed that gallotannin inhibited the rTBAK with a *K*_*i*_ value of 6.3 nM when L-arginine was the variable substrate. With ATP as the variable substrate, however, the *K*_*i*_ value was 1.04 nM.

Analysis using Hanes Woolf and/or linear regression model revealed that inhibition by gallotannin may not be competitive. The affinity of rTbAK for its substrates was not affected. The *K*_*m*_ of rTbAK for the substrates remain largely unaffected. In contrast, sharp reduction in the maximal velocity of the rTbAK enzyme suggests noncompetitive interactions.

### 2.2. Fluorescence Analysis

**Figure 3:**
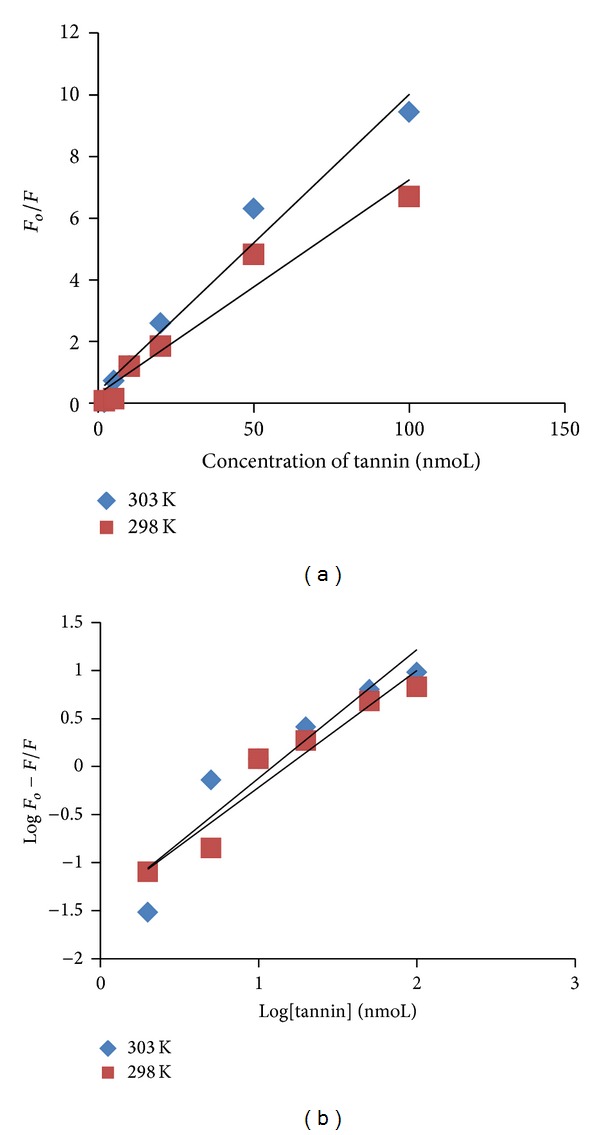
Stern-Volmer plots showing fluorescence quenching of the recombinant arginine kinase of* Trypanosoma brucei* in the presence of gallotannin at different temperatures. (a) Quenching of arginine kinase of* Trypanosoma brucei* by gallotannin and (b) Quenching of arginine kinase of* Trypanosoma brucei* by gallotannin.

Fluorescence spectra were obtained by keeping the concentration of rTbAK constant, while varying the concentrations of nanoparticles and gallotannin at *λ*_ex_ 295 nm [13]. Increasing concentrations of gallotannin led to an appreciable reduction in rTbAK fluorescence. To determine whether quenching by nanoparticles or gallotannin was static or dynamic, the Stern-Volmer plots at different temperatures (298 and 303 K) were made (Figure 3) based on (2). The *K*_sv_ decreased with increasing temperature (Table 1), suggesting a static quenching by gallotannin.

Binding constant and stoichiometry of binding sites on rTbAK were determined based on (3).

The plots of log⁡ (*F*_*o*_ − *F*/*F*)  versus log⁡ [*Q*] based on (4) for gallotannin-rTbAK system at different temperatures and results are shown in Figure 3. The values of *K* and *n* derived from the intercepts and slope of these plots are listed in Table 2. The *K* values suggested a strong interaction between gallotannin and rTbAK. The *n* value approximately equals 1 suggesting that gallotannin may have quenched fluorescence by binding at the Trp site. The rTbAK has two Trp residues at positions 226 and 243 separated by 16 amino acid residues. In order to gain more insights into the interactions between gallotannin and the rTbAK, thermodynamic parameters were calculated from Van't Hoff plots. Changes in both enthalpy (Δ*H*_*o*_) and entropy (Δ*S*_*o*_) were obtained using (6) and (7).

Based on the values of *K* at different temperatures, changes in free energy (Δ*G*_*o*_), enthalpy (Δ*H*_*o*_), and entropy (Δ*S*_*o*_) were obtained using (5) and (6).

**Figure 4:**
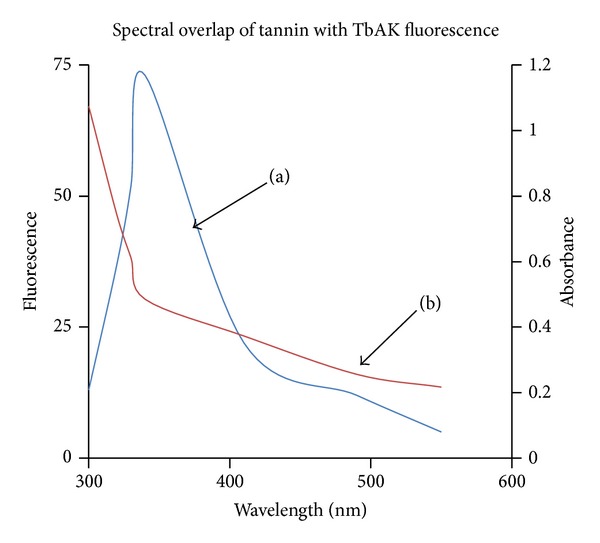
Spectral overlap between the fluorescence of arginine kinase of* Trypanosoma brucei* and the absorbance of gallotannin. (a) Fluorescence emission spectrum of rTbAK and (b) UV spectrum of gallotannin.

The positive values of free energy (Δ*G*_*o*_) indicated a nonspontaneous reaction. However, negative enthalpy (Δ*H*_*o*_) and positive entropy (Δ*S*_*o*_) values suggested hydrophobic interaction between gallotannin and rTbAK. Furthermore, fluorescence analysis revealed spectral overlap between the fluorescence emission spectrum of free rTbAK and the UV absorption spectra of gallotannin (Figure 4).

Fluorescence resonance energy transfer (FRET) analysis was applied to determine the distance between the nanoparticles or tannin as acceptor and rTbAK as energy donor in the interaction resulting in fluorescence quenching. The existence of spectral overlap between the fluorescence emission spectrum of free rTbAK and the UV absorption spectra of gallotannin (Figure 4) was a basis for the distance *r* between these species to be calculated from Forster's theory as efficiency of energy transfer, *E* (8). *R*_*o*_ is the critical distance at which the energy transfer is 50% and was estimated using (9). The spectral overlap integral (*J*) between the donor emission spectrum and the acceptor absorbance spectrum was estimated based on (10). The overlap integral was calculated by integrating the overlap area of the spectrum. The values for *J*, *R*_*o*_, and *r* are as shown in Table 3.

## 3. Discussion

Following the biochemical and molecular characterisation of arginine kinase (AK) in* trypanosomatids* [2, 3], subsequent studies have demonstrated that the AK enzyme could be a novel target in the development of new trypanocides [4, 5, 9]. Accordingly, specific or selective inhibitors for arginine kinase of the* trypanosomatids* are desirable.

The current study investigated the interaction between gallotannin and recombinant arginine kinase of* T. brucei* (rTbAK). Data presented implicate inhibitory interaction between gallotannin and rTbAK as well as the quenching of rTBAK's fluorescence intensity. Gallotannin reduced the activity of the rTbAK to less than 30%, which is consistent with previous reports on the inhibitory potential of phenolic-related compounds on AK activity [14, 15]. Further kinetic analysis suggests that inhibition by gallotannin may be noncompetitive. The *K*_*m*_ values for the rTbAK substrates (L-arginine and ATP) remained fairly constant. Rather, the *V*_max⁡_ of the enzyme was reduced in the presence of the gallotannin. When the L-arginine was variable substrate, an approximate >84% decrease was recorded in *V*_max⁡_. With ATP as variable substrate, however, >97% decrease in *V*_max⁡_ of rTBAK was caused by gallotannin. The respective low *K*_*i*_ values obtained at varied concentrations of either L-arginine or ATP underscore the strong affinity of gallotannin for rTBAK. Earlier studies have demonstrated the inhibitory potential of flavonoids on the activity of arginine kinase [14, 15]. In these studies, effect of flavonoids or rutin on the arginine kinase enzyme was found to correspond to noncompetitive inhibition. The fact is clearly supported in the present study, except that the inhibition by gallotannin was not concentration dependent. The affinity of rTbAK for its substrates was not affected. The *K*_*m*_ of rTbAK for the substrates remain largely unaffected. In contrast, sharp reduction in the maximal velocity of the rTbAK enzyme suggests noncompetitive interactions.

The thermodynamic data were estimated for the inhibition of the rTbAK activity by gallotannin at different temperatures and the results demonstrated the involvement of hydrophobic interactions. The positive values of the free energy (Δ*G*_*o*_) may suggest nonspontaneous reaction. However, the negative and positive enthalpy and entropy, respectively, are consistent with hydrophobic force interaction between gallotannin and rTbAK [16]. On the other hand, the binding constants and number of binding sites obtained from the plots of log⁡ (*F*_*o*_ − *F*/*F*)  versus log⁡ [*Q*] correspond to strong interaction between the rTbAK and gallotannin. The value of *n* (stoichiometry of binding) approximates 1 implicating the formation of gallotannin-rTbAK complexes. The formation of such complexes may help explain the quenching of fluorescence intensity of rTbAK by gallotannin (Figure 3). The *K* values suggested a strong interaction between gallotannin and rTbAK. Although the mechanism of inhibition is yet unclear, it could be inferred that the fluorescence quenching by gallotannin may be near one of the Trp residue sites. Furthermore, fluorescence analysis revealed spectral overlap between the fluorescence emission spectrum of free rTbAK and the UV absorption spectra of gallotannin, suggesting possible occurrence of energy transfer between the interacting species [17]. The *K*_sv_ values which decreased with increasing temperature and the data in Table 1 may indicate that the fluorescence quenching interaction between gallotannin and rTBAK was static [18, 19].

The values for *J*, *R*_*o*_, and *r* obtained in this study are consistent with conditions of Forster energy transfer theory. It has been demonstrated that value for this distance (*r*) that is less than 10 nm and between 0.5 and 1.5 of the *R*_*o*_ value indicates a high probability that fluorescence transfer is taking place [10].

The FRET analysis revealed the *r* values to be greater than the *R*_*o*_ values. This suggests occurrence of energy transfer between the rTbAK and the gallotannin. The FRET analysis revealed that the *r* value is greater than the *R*_*o*_ value; this supports further the submission that static quenching interactions existed between gallotannin and the rTbAK [18, 20]. More so, the FRET analysis showed that the *r* value (distance between the gallotannin and the rTbAK enzyme) is greater than the *R*_*o*_ (Forster critical distance) value.

## 4. Conclusion

Data revealed inhibitory interactions between gallotannin and rTbAK. To our knowledge, this is the first report demonstrating the inhibitory potentials of gallotannin on the activity of rTbAK. The inhibitory relationships were consistent with noncompetitive interactions. Furthermore, the Stern-Volmer plots indicated that interactions involving the gallotannin, which caused the quenching of fluorescence intensity of the rTbAK, may be static. This is supported by the FRET analysis which also implicates the high probability for fluorescence exchange between rTBAK and gallotannin. Although the mechanism of inhibition is not clear yet, evidence presented warrants further kinetic analysis including molecular docking in order to clearly define the inhibitory interactions between the gallotannin and rTbAK. The insights of the binding properties have become necessary if selective inhibition of TbAK and eventual better trypanocides are desirable.

## 5. Materials and Methods

All reagents were of analytical grade and obtained from Sigma, USA, unless otherwise stated.

### 5.1. The Recombinant Form of the Arginine Kinase of* Trypanosoma brucei*

The rTBAK was obtained from the Nanomedicine & Biomedical Target Laboratory, Department of Biochemistry, Microbiology and Biotechnology, Rhodes University, South Africa. The cloning, expression, purification, and characterization have been previously reported in earlier studies [10, 21].

### 5.2. Protein Determination and Assay of Activity for Arginine Kinase of* Trypanosoma brucei* (rTbAK)

Total protein concentration was determined using the Bradford protocol. The activity of rTbAK was assayed using a previously described protocol [10]. Briefly, 100 mM Tris-HCl buffer (pH 8.6) containing 10 mM L-arginine, 5 Mm ATP disodium salt, 10 mM mercaptoethanol, and 200 mM magnesium sulfate was added to 20 *μ*L (0.002 mmol final concentration) of the enzyme. The reaction mixture was incubated for 5 minutes at 30°C and stopped by the addition of 2.5% TCA. This was heated at 100°C in heating block and cooled on ice for 2 minutes. The inorganic phosphate (pi) determination reagent (L-ascorbic acid and ammonium molybdate) was added and absorbance reads at 700 nm, using the Synergy Mx, (Monochromator Multi-Mode Microplate Reader, Biotek Instruments, Inc., USA). The control assay samples lacked the enzyme. Calibration curve was constructed to correlate the absorbance change with the generation of inorganic phosphate (pi). One unit of rTbAK activity is the amount of enzyme that catalyses the formation of 1 *μ*mol inorganic phosphate per minute per mL.

### 5.3. Estimation of Kinetic Parameters for the Interaction between Gallotannin and the Recombinant Arginine Kinase of* Trypanosoma brucei* (rTbAK) Protein

Assays were carried out as earlier described for protein and enzyme assay determination [10, 21]. The kinetic parameters for the interaction of gallotannin with rTbAK were determined by including the gallotannin at a concentration of 10–50 nM with either arginine, between 0.1 and 2 mM concentration at fixed level of ATP (0.5 mM), or ATP, between 0.1 and 0.5 mM concentration at fixed level of arginine (2 mM). One unit of rTbAK activity is the amount of enzyme that catalyses the formation of 1 *μ*mol inorganic phosphate per minute per mL. Furthermore, the fluorescence resonance energy transfer (FRET) study and estimation of thermodynamic parameters were conducted to evaluate the interaction between the rTBAK and gallotannin at two different temperatures 298 and 303 K using the Synergy Mx (Monochromator Multi-Mode Microplate Reader, Biotek Instruments, Inc., USA).

In order to evaluate the binding affinity of rTBAK for gallotannin, the inhibitor constant (*K*_*i*_) was estimated using

(1) $$\begin{array}{c} K_{i}=\frac{\left[\text{GT}\cdot {V_{\max}}^{\text{app}}\right]}{\left[V_{\max}-{V_{\max}}^{\text{app}}\right]}, \end{array}$$

where GT is the concentration of gallotannin and *V*_max⁡_^app^ is the apparent maximum velocity in the presence of gallotannin.

### 5.4. Fluorescence Analysis

Fluorescence spectra were obtained by keeping the concentration of rTbAK constant, while varying the concentrations of nanoparticles and gallotannin at *λ*_ex_ 295 nm [13]. Increasing concentrations of gallotannin led to appreciable reduction in rTbAK fluorescence. To determine whether quenching by nanoparticles or gallotannin was static or dynamic, the Stern-Volmer plots at different temperatures (298 and 303 K) were made based on the following equations [22]:

(2) $$\begin{array}{c} \frac{F_{o}}{F}=1+K_{\text{sv}}\left[Q\right], \end{array}$$

where *F*_*o*_ and *F* are the fluorescence intensities in the absence and presence of the quencher, respectively. *Q* is the quencher and herein refers to gallotannin. *K*_sv_ is the Stern-Volmer quenching constant.

Binding constant and stoichiometry of binding sites on rTbAK were determined following this equation:

(3) $$\begin{array}{c} nQ+B=Q_{n}B,\\ K=\frac{\left[Q_{n}B\right]}{{\left[Q\right]}^{n}\left[B\right]}=\frac{\left[B_{o}\right]-\left[B\right]}{{\left[Q\right]}^{n}\left[B\right]}, \end{array}$$

where *K* refers to the binding constant and *n* refers to the number of binding sites; [*Q*] and [*B*] are the concentrations of quencher and protein, while [*Q*_*n*_ *B*] is the concentration of nonfluorescent complex of quencher protein. Total concentration of protein is [*B*_*o*_]. The fluorescent intensity is proportional to the concentration of protein given as

(4) $$\begin{array}{c} \frac{\left[B\right]}{\left[B_{o}\right]}=\frac{F}{F_{o}}. \end{array}$$

From (3), it can further be deduced as [14]

(5) $$\begin{array}{c} \log\left(\frac{F_{o}-F}{F}\right)=\log K+n\log\left[Q\right]. \end{array}$$

The plots of log⁡ (*F*_*o*_ − *F*/*F*)  versus log⁡ [*Q*] were based on (4) for gallotannin-rTbAK system at different temperatures. In order to gain more insights into the interactions between gallotannin and the rTbAK, thermodynamic parameters were calculated from Van't Hoff plots. Changes in both enthalpy (Δ*H*_*o*_) and entropy (Δ*S*_*o*_) were obtained from the following equations [15, 23]:

(6) $$\begin{array}{c} \Delta G_{o}=-\text{RT}\;\ln K=\Delta H_{o}-T\Delta S_{o}, \end{array}$$

(7) $$\begin{array}{c} \ln\frac{K2}{K2}=\left(\frac{1}{T1}-\frac{1}{T2}\right)\frac{\Delta H_{o}}{R}. \end{array}$$

Based on the values of *K* at different temperatures, changes in free energy (Δ*G*_*o*_), enthalpy (Δ*H*_*o*_), and entropy (Δ*S*_*o*_) were obtained using (5) and (6).

Fluorescence resonance energy transfer (FRET) analysis was applied to determine the distance between the nanoparticles or tannin as acceptor and rTbAK as energy donor in the interaction resulting in fluorescence quenching. The existence of spectral overlap between the fluorescence emission spectrum of free rTbAK and the UV absorption spectra of gallotannin was a basis for the distance *r* between these species to be calculated from Forster's theory [17] in which the efficiency of energy transfer, *E*, is given by

(8) $$\begin{array}{c} E=1-\frac{F}{F_{o}}=\frac{{R_{o}}^{6}}{{R_{o}}^{6}+r^{6}}. \end{array}$$

Here, *r* refers to the distance between nanoparticles or tannin and Trp residue in TbAK. *F* and *F*_*o*_ are the fluorescence intensities in the presence and absence of gallotannin. *R*_*o*_ is the critical distance at which the energy transfer is 50%. Consider

(9) $$\begin{array}{c} {R_{o}}^{6}=0.2108\;K^{2}N^{-4}\Phi J. \end{array}$$

Here, *N* is the refractive index of the medium. *K* ^2^ is the orientation factor. Φ is the quantum yield of donor. Under the present experimental conditions, *K* ^2^ = 2/3, Φ = 0.118, and *N* = 1.336 [24]. The spectral overlap integral (*J*) between the donor emission spectrum and the acceptor absorbance spectrum was estimated by the following summation:

(10) $$\begin{array}{c} J=\int f_{D}\left(\lambda \right)\epsilon_{A}\left(\lambda \right)\lambda^{4}d\lambda , \end{array}$$

where *f*_*D*_ is the normalized donor emission spectrum and *ϵ*_*A*_ is the molar extinction of acceptor. The overlap integral was calculated by integrating the overlap area of the spectrum.

### 5.5. Data Analysis

All analyses were carried out in triplicate and values reported as the means with standard error of mean significant at *P* < 0.05 versus controls, where necessary analysis of variance was conducted using the GraphPad Prism for Windows (GraphPad Prism Software Inc.) and Microsoft Excel 2007.

## Figures and Tables

**Table 1:** Stern-Volmer constant (*K*_sv_) estimated for the interaction between recombinant arginine kinase of *Trypanosoma brucei* (rTbAK) and gallotannin.

Inhibitors	*T* (K)	*K*_sv_ (L mol^−1^)	*R* ^a^
Gallotannin	298	11.10 × 10^4^	0.9246
	303	10.00 × 10^4^	0.8828

^a^The correlation coefficient.

**Table 2:** Thermodynamic parameters, binding constant (*K*), and number of binding sites (*n*) estimated for the interaction between recombinant arginine kinase of *Trypanosoma brucei* (rTbAK) and gallotannin.

Inhibitors	*T* (K)	*K* (L mol^−1^)	*n*	*R* ^a^	Δ*H*_*o*_ (KJ/mol)	Δ*G*_*o*_ (KJ/mol)	Δ*S*_*o*_ (KJ/mol)
Gallotannin	298	3.7 × 10^4^	1.2176	0.9494	−8.40	8.12	55.60
	303	3.5 × 10^4^	1.3381	0.9713		8.45	55.60

^a^The correlation coefficient.

**Table 3:** Estimates of the distance (*r*) for the interaction between arginine kinase of *Trypanosoma brucei* (TbAK) and gallotannin.

Inhibitors	Spectral overlap (*J*) cm^3^ L mol^−1^	*R*_*o*_ (nm)	*r* (nm)
Gallotannin	4.33 × 10^−14^	2.13	2.28
