# Corrigendum to “Interaction between Gallotannin and a Recombinant Form of Arginine Kinase of *Trypanosoma brucei*: Thermodynamic and Spectrofluorimetric Evaluation”

**DOI:** 10.1155/2018/1914746

**Published:** 2018-09-06

**Authors:** O. S. Adeyemi, A. F. Sulaiman, O. M. Iniaghe

**Affiliations:** ^1^Department of Biological Sciences, Landmark University, Omu-Aran 370102, Nigeria; ^2^Department of Biochemistry, Faculty of Life Sciences, University of Ilorin, Ilorin 240001, Nigeria; ^3^Department of Biochemistry, Ambrose Alli University, Ekpoma 310001, Nigeria

In the article titled “Interaction between Gallotannin and a Recombinant Form of Arginine Kinase of* Trypanosoma brucei*: Thermodynamic and Spectrofluorimetric Evaluation” [1], there were a typo in Equation (7), where the denominator K2 should be corrected to K1, and an error in Table 2, where the values for* Δ* **H,** *Δ* **S,** and* Δ* **G** were incorrectly calculated. The equation and table are corrected as follows:

(7) $$\begin{array}{c} \ln\frac{k2}{k1}=\left(\;\frac{1}{T1}–\frac{1}{T2}\;\right)\frac{\Delta H_{o}}{R}. \end{array}$$

## Figures and Tables

**Table 2:** Thermodynamic parameters, binding constant (K), and number of binding sites (n) estimated for the interaction between arginine kinase of *Trypanosoma brucei* (TbAK) and gallotannin.

**Inhibitors**	**T (K)**	**K (Lmol** ^**-1**^ **)**	**n**	**R** ^**a**^	Δ**H**_**o**_ **(KJ/mol)**	Δ**G**_**o**_ **(KJ/K/mol)**	Δ**S**_**o**_ **(J/mol/K)**
Tannin	298	3.7 × 10^4^	1.2176	0.9494	-8.40	-26.06	59.26
	303	3.5 × 10^4^	1.3381	0.9713		-26.35	59.24

**a is the correlation coefficient.**
